# Factors of Negative Affect in Elderly Patients With Substance Use Disorders During COVID-19 Pandemic

**DOI:** 10.3389/fpsyt.2021.697472

**Published:** 2021-07-20

**Authors:** Qianjin Wang, Yingying Wang, Jinsong Zuo, Yanan Zhou, Winson Fu Zun Yang, Yanhui Liao, Jinsong Tang, Xin Wang, Qian Yang, Qiuxia Wu, Hanjing Emily Wu, Colin B Goodman, Tieqiao Liu, Xiangyang Zhang

**Affiliations:** ^1^National Clinical Research Center for Mental Disorders, and Department of Psychiatry, the Second Xiangya Hospital of Central South University, Changsha, China; ^2^Hunan Key Laboratory of Psychiatry and Mental Health, Changsha, China; ^3^School of Life Science and Chemistry, Hunan University of Technology, Zhuzhou, China; ^4^Department of Psychological Sciences, Texas Tech University, Lubbock, TX, United States; ^5^Department of Psychiatry, Sir Run Run Shaw Hospital, School of Medicine, Zhejiang University, Hangzhou, China; ^6^Department of Psychiatry and Behavioral Sciences, the University of Texas Health Science Center at Houston, Houston, TX, United States; ^7^CAS Key Laboratory of Mental Health, Institute of Psychology, Chinese Academy of Sciences, Beijing, China

**Keywords:** substance use disorders, negative affect, elderly, impulsivity, cravings, COVID-19

## Abstract

**Background:** The outbreak of the novel coronavirus disease 2019 (COVID-19) has become the greatest public health emergency and has attracted global attention. During the COVID-19 pandemic, the negative affect (NA) of elderly patients with substance use disorders (SUDs) has also become a more serious public concern. The current study aims to clarify the NA and its influencing factors in elderly patients with SUDs during the pandemic.

**Methods:** Two psychiatrists conducted semi-structured interviews with 77 SUD patients aged above 50 years to collect their demographical information and certain drug use characteristics. Barratt Impulse Scale and the Positive and Negative Affect Scale were used to obtain information about patients' self-reported impulsivity and NA.

**Results:** Univariate linear regression analysis showed that NA was positively correlated with the frequency of drug use, type of SUDs, cravings during COVID-19, and impulsivity. Multiple linear regression analysis showed that being female, higher frequency of drug use, stronger cravings, and greater impulsiveness jointly accounted for the variation of NA in elderly patients with SUDs.

**Conclusions:** This study confirmed that, during the COVID-19 pandemic, gender, frequency of drug use, cravings, and impulsivity were associated with NA in elderly patients with SUDs. This study provided a theoretical basis for clinicians to reduce the patients' NA.

## Introduction

The outbreak of the novel coronavirus disease 2019 (COVID-19) has become the greatest public health emergency and has attracted global attention (1). Although the case fatality rate of COVID-19 is relatively low compared with the SARS virus outbreak in 2003 and Ebola virus outbreak in 2014, it inevitably leads to more serious public panic because of its easier spread, widespread uncontrollability, and uncertainty about the incubation period of the virus (2, 3). Excessive misinformation on social media and unprecedented large-scale quarantine measures that basically limited residents to their homes have undoubtedly exacerbated the panic (4, 5). Therefore, the COVID-19 pandemic has been a stressor for millions of people (6, 7). As we all know, most stress events will impact people's physical and mental health in some way, pose a serious threat to people's mental health, and subsequently lead to negative affect (NA) such as anxiety and depression (8, 9). Emerging evidences suggested that the pandemic has exacerbated substance use and mental health symptoms in the most vulnerable populations (10). Especially for children (11), the elderlies (12), and patients with mental illness (13, 14), COVID-19 has been a heavy blow to their fragile psychological endurance capability.

As mentioned above, the psychological vulnerability had led to more NA for the elderlies during the pandemic (15). In particular, clinicians have conducted extensive studies on elderly patients with mood disorder (16, 17) and dementia (18) during the pandemic, as these illnesses are often identified as severe mental disorders (19). Undoubtedly, these studies provided guidance for clinicians to treat such patients in a more targeted manner, thus helped them positively cope with their NA. However, for the elderlies with substance use disorders (SUDs), it is obvious that their mental and psychological problems are rarely considered by clinicians and researchers before their physical symptoms are addressed. However, studies have shown that the mental and psychological problems of patients with SUDs might relapse or be exacerbated by social isolation and lockdown during a pandemic (20–22). Patients with previous SUDs are at greater risk of adverse consequences after contracting COVID-19 (23). To sum up, these patients are more likely to have mental and psychological problems in the face of a pandemic, which prompts clinicians to pay more attention to their mental and psychological problems while caring about their physical symptoms.

In view of the potential threat of NA, studies on affectivity associated with the pandemic have been carried out, which provided basis for experts to follow closely on mental health services during the pandemic. However, these studies are focused on the general population (7, 14) and did not provide evidence on the role NA has been playing in the prognosis and relapse in elderly with SUDs during the COVID-19 pandemic (24). Moreover, lower NA can effectively reduce drug use during medical treatment (25, 26) and cravings for various substances (e.g., cigarettes, cocaine, opiates, and alcohol) (27) and further contributes to the sustainable withdrawal from additive substances in elderlies with SUDs after leaving treatment discontinuation (28). Therefore, it is critical to address the issue of NA in response to the COVID-19 pandemic (29). Therefore, it is urgent for clinicians to gain an understanding of factors leading to NA in elderlies with SUDs under the dual pressures of pandemic and forced withdrawal in order to improve patients' NA in an economical and effective way.

According to some previous studies, impulsivity is associated with NA (30, 31), especially in patients with impulsive mental disorders, such as bipolar disorder (32) and borderline personality disorder (33). In the current popular diagnostic systems, such as Diagnostic and Statistical Manual of Mental Disorders, Fifth Edition (DSM-5) and International Classification of Diseases, Eleven Edition (ICD-11), although impulsivity is not the core symptom of these mental disorders, certain impulsive behaviors can still be used to identify SUDs, such as uncontrolled drug seeking (34). Therefore, we hypothesized that under the dual pressure of the pandemic and forced withdrawal, high impulsivity may be related to NA. Unfortunately, few studies were conducted on the relationship between impulsivity and NA regarding SUDs during the COVID-19 pandemic. In addition to its relationship with NA, as confirmed by previous studies (35, 36), cravings is also the core symptom of SUDs and plays an important role in the diagnosis of this disorders (37). Hence, we also assumed that cravings under the dual pressure of the pandemic and forced withdrawal may be related to NA. However, the current studies on cravings and NA are based on tobacco and alcohol consumption (38, 39), and there still lacks evidence regarding cravings and NA in the elderly population with SUDs. In addition to the above mentioned clinical variables, there are other factors related to NA in this population, with the most common ones being characteristics related to drug use, including the frequency and duration (40, 41). In this study, we aim to elucidate the relationship between these clinical variables and NA, especially to determine to what extent the impulsivity, cravings, and other characteristics of substance use explain the variations in NA in elderlies with SUDs.

## Methods and Materials

### Participants

The study was organized by the Second Xiangya Hospital of Central South University as an investigation of psychology and characteristics of substance use during COVID-19. Since March 2020, 77 patients with SUDs aged over 50 years have been recruited from two compulsory drug rehabilitation centers in Changsha, Hunan Province. Of the 77 patients, 22 were users of new drug abusers (e.g., methamphetamine/ketamine) and 55 were users of traditional drug abusers (heroin). All the subjects were evaluated by two trained and experienced psychiatrists *via* semi-structured interviews, and the consistency of the two scores was as high as 95%. The inclusion/exclusion criteria of this study are as follows: (1) all subjects must meet the diagnostic criteria for SUDs of the DSM-5; (2) all subjects must be aged ≥50 years; (3) all subjects must have normal intelligence and cognitive functions; (4) all subjects must have no previous or current mental illness or family history of mental illness; (5) all subjects must have no alcohol use disorder; (6) all subjects must have no other serious disease that conforms to DSM-5 or ICD-10.

This study was approved by the Ethics Committee of the Second Xiangya Hospital of Central South University and conducted in accordance with the Helsinki Declaration. All the subjects signed the informed consent after fully informed about the purpose, process, benefits, and risks of the study, and voluntarily participated in this study. All data and patient information were kept confidential throughout the study.

### Clinical Assessment

All the subjects completed the following self-report scales; all the instruments have good reliability and validity.

#### Demographic Data and Drug Use Characteristics

Demographic information included age (elderly subjects aged 50 and above), gender, education, marital status, employment status, and income. The characteristics of drug use include duration (year), frequency, and types of drugs use (i.e., new drugs including methamphetamine and ketamine and traditional drugs including heroin).

#### Cravings

Cravings of the subjects were measured using the Visual analog scale (VAS), which is a psychometric response scale with 10 graduations, with 0 indicating no craving and 10 indicating extreme craving (42, 43). This scale has been widely used in measuring the drug cravings with high reliability (44, 45). During the assessment, the participants were required to draw a marker on a horizontal line to indicate their current cravings for drugs.

#### Impulsivity

The degree of impulsivity was measured using the Barratt impulse scale (BIS), which is the most extensive self-report scale for this purpose (46). The Chinese version of BIS-11 was used to measure the cognitive impulsiveness, motor impulsiveness, and unplanning impulsiveness of SUDs; among the subscales, items in the motor impulsiveness subscale were balanced positively, while the cognitive impulsiveness and the unplanning impulsiveness subscale used a reverse scoring (31). The whole scale consists of 30 items, using a 5-point Likert scale for each item; higher total score indicated stronger impulsiveness (47). In this study, the Cronbach's α of the whole scale was 0.909.

#### Negative Affect

The NA of subjects was measured using a 10-item subscale of the Positive and Negative Affect Scale (PANAS) (48). In this scale, each item was rated from 1 (not at all) to 5 (extremely severe), with the total score ranging from 10 to 50 (49); higher total score indicated more obvious NA (50). In this study, the internal consistency of the NA subscale was 0.83. The NA of the subjects in the past week was measured (49).

### Statistical Analysis

SPSS for Windows (Version 24, SPSS Inc., Chicago, IL, USA) software package was used for statistical analysis. Prior to the analyses, normality of data distribution on each variable was tested using the Kolmogorov–Smirnov test. Demographic and drug use characteristics were presented using descriptive data. Univariate linear regression analysis was used to initially identify the relationship between impulsivity, craving, drug use characteristics, and NA, and multiple linear regression analysis was used to further examine the influence of the above variables on NA. A regression model was established with NA as the dependent variable and the index of *p* < 0.1 in the univariate regression analysis as the independent variable. The threshold of statistical significance was set at *p* < 0.05 (two-tailed).

## Results

### Demographics Characteristics

Demographic information of the patients are shown in Table 1. Of the 85 patients with SUDs over the age of 50 who were invited to participate in the survey, 77 completed the questionnaire, including 11 females (14.3%) and 66 males (85.7%). The typical feature of the entire sample group is their socioeconomic status, which was of the middle class. The average age of the patients was 53.95 ± 3.73 years. Among the patients, 29.9% had full-time jobs and 59.7% had unstable incomes.

**Table 1:** Demographic information of the subjects with substance use disorders (*N* = 77).

**Variables**	**M ± SD**	***N* (%)**
**Gender**		
Male		66 (85.7)
Female		11 (14.3)
**Age (year)**	53.95 ± 3.73	
**Education (year)**	9.29 ± 3.03	
**Marital status**		
Married		35 (45.5)
Unmarried/divorced		42 (54.5)
**Employment status**		
Full time		23 (29.9)
Part-time/unemployed		54 (70.1)
**Income (CNY)**		
Stable		31 (40.3)
Unstable		46 (59.7)

*M, mean; SD, standard deviation; n, number; %, the percentage of participants; CNY, Chinese Yuan*.

### Drug Use Characteristics, Cravings, Total BSI-11 Score and NA

All the participants met the criteria for substance dependence in the DSM-5; of all the patients, 21 (27.3%) were diagnosed with methamphetamine use disorder, 1 (1.3%) was diagnosed with ketamine use disorder, and 55 (71.4%) were diagnosed with heroin use disorder. Their substance use characteristics are reported in Table 2.

**Table 2:** Drug use characteristics, cravings, total score of BIS-11, and NA (*N* = 77).

**Variables**	**M ± SD**	***N* (%)**
**Types of drug use**		
New drugs		19 (24.7)
Traditional drugs		58 (75.3)
**Duration of drug use (year)**	23.59 ± 9.30	
**Frequency of drug use**	2.57 ± 1.19	
**Cravings**	3.97 ± 3.19	
**Total score of BIS-11 (0.882)**^**a**^	145.81 ± 35.18	
**Total score of NA (0.875)**^**a**^	24.44 ± 7.02	

*M, mean; SD, standard deviation; n, number; %, the percentage of participants; BIS-11, Barratt impulse scale-11; NA, negative affect; a, Cronbach's α*.

### Relationship Between Drug Use Characteristics, Cravings, Impulsivity, and NA

**Figure 1:**
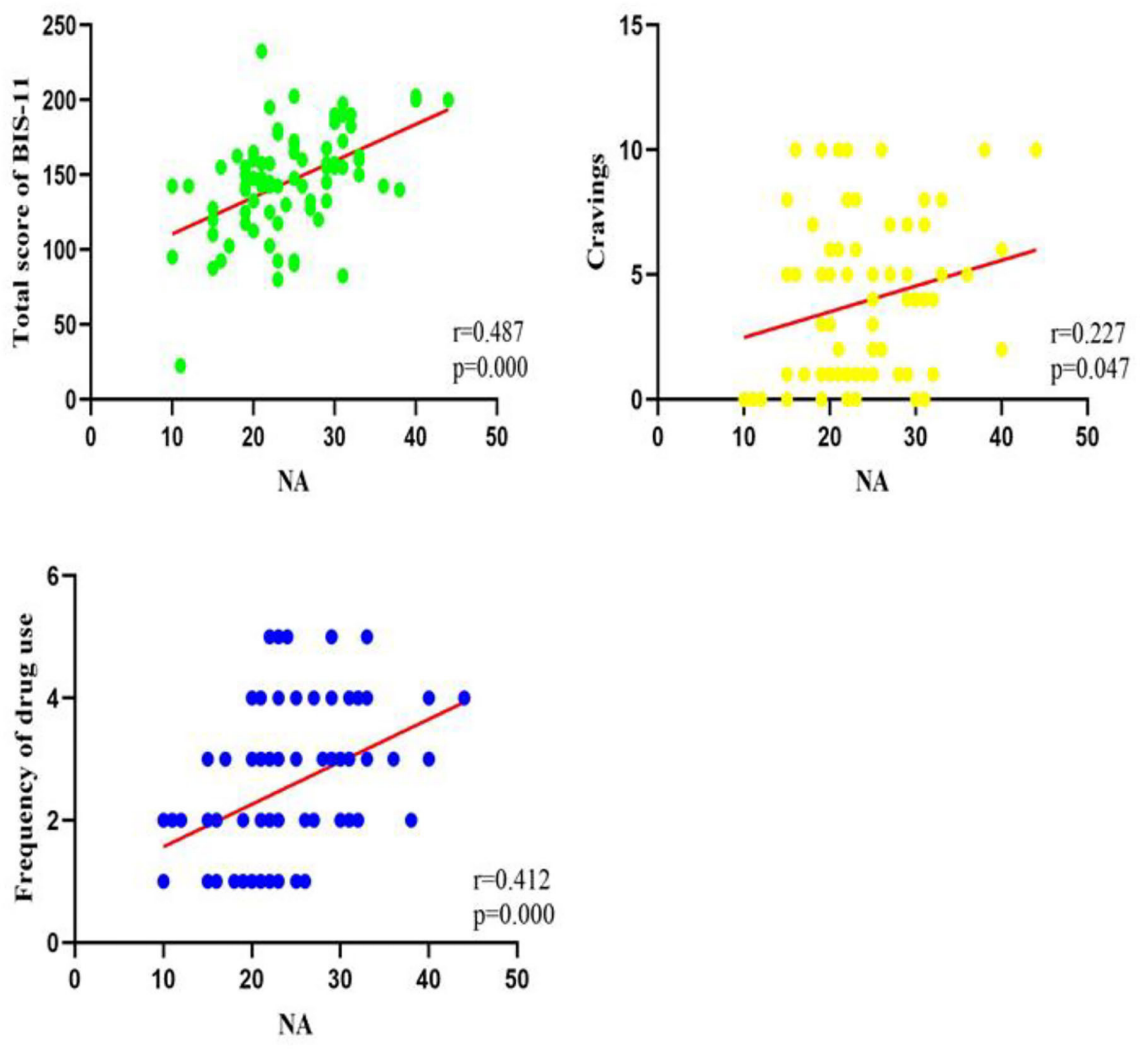
Correlations of total score of BIS-11, cravings, frequency of drug use, and total score of NA (*N* = 77).

Univariate linear regression analysis was performed between the total score of NA and drug use characteristics, cravings, and the total score of BIS-11. The NA total score was positively correlated with drug use frequency (*r* = 0.41, *p* < 0.001), types of drug use (*r* = 0.29, *p* = 0.010), cravings (*r* = 0.23, *p* = 0.047), and the BIS-11 total score (*r* = 0.49, *p* < 0.001). There was no significant correlation between the NA score and the duration of drug use (*r* = 0.19, *p* = 0.096). The results of correlation results are shown in Table 3 and Figure 1.

**Table 3:** Univariate regression of drug use characteristics, cravings, impulsivity, and NA.

		**Duration of drug use**	**Frequency of drug use**	**Cravings**	**Total score of BIS-11**	**Types of drug use**
**Total score of NA**	**β**	0.191	0.412	0.227	0.487	0.291
	***p***	0.096	0.000	0.047	0.000	0.010

*BIS-11, Barratt impulse scale-11; NA, negative affect*.

### Multiple Linear Regression of Age, Clinicals Variables, Impulsivity, and NA

Multiple linear regression analysis was conducted to examine the relationship between age, gender, education, marital status, employment status, income, duration of drug use, frequency of drug use, types of drug use, cravings, BIS-11, and NA. It was found that gender, drug use frequency, cravings, and BIS-11 total score could jointly account for the variation of NA. In other words, being female, long-term drug use, greater drug cravings, and impulsiveness were associated with more NA (Table 4).

**Table 4:** Multiple linear regression of all the variables in this study.

**Variables**	**B**	**β**	**T**	***p***
**Age**	0.05	0.03	0.29	0.77
**Gender**	5.30	0.27	2.78	0.007
**Education**	−0.22	−0.10	−0.92	0.36
**Marital status**	0.05	0.004	0.04	0.97
**Employment status**	1.41	0.09	0.91	0.37
**Income**	0.63	0.04	0.47	0.64
**Duration of drug use**	−0.01	−0.01	−0.06	0.96
**Types of drug use**	1.55	0.10	0.96	0.34
**Frequency of drug use**	2.37	0.40	4.02	0.000
**Cravings**	0.57	0.26	2.57	0.012
**Total score of BIS-11**	0.06	0.30	3.13	0.003

*BIS-11, Barratt impulse scale-11*.

## Discussion

To our knowledge, this is the first study to explore negative affect and related factors in patients with SUDs aged 50 and older during the COVID-19 pandemic. The main findings are as follows: first, some demographic characteristics (gender), drug use characteristics (frequency of drug use), cravings, and impulsivity are related to NA in these elderly patients; second, the NA of the elderly patients with SUDs was positively correlated with gender, drug use frequency, cravings, and impulsivity; and finally, gender, drug use frequency, cravings, and impulsivity jointly explain the variations of NA in elderly patients with SUDs.

Regarding demographic information, gender can be used as a predictor of NA during the pandemic. Specifically, females with SUDs are more likely to have NA. Previous studies on SUDs (51, 52) and other mental disorders (53, 54) have consistently shown that females are more susceptible to NA when faced with unique stress experiences brought about by catastrophic events such as SARS and earthquakes (21, 55, 56), and that greater NA is associated with greater emotional regulation disorders and is associated with affective, anxiety, and SUDs (57). Studies also showed that among patients with SUDs, women generally develop addictions faster than men and are more likely to have concurrent mental disorders, supporting the theory that substance use is a coping strategy for many women (24). With fewer opportunity to access previously cultivated supportive relationships due to social isolation caused by the lockdown during the pandemic, women may feel more isolated and thus have more NA, as they might depend more on social supports (6). Our results also indirectly confirmed that women may have more NA when they have stressful experience, which was likely to lead to higher frequency of drug. Therefore, clinical workers and relevant researchers need to pay more attention to such phenomenon and provide female patients more psychological care and counseling.

For characteristics of drug use, we found that NA was positively correlated with the frequency of drug use during the pandemic, i.e., higher frequency of substance use indicated more NA experience. This result is also consistent with the results of most previous studies, which have shown that higher frequency of drug use is closely associated with the occurrence of NA (such as anxiety and depression) (40, 58). Moreover, the COVID-19 pandemic increased people's vulnerability to SUDs, which in turn contributed to higher NA in patients who developed SUDs (23). Compared with patients with a lower frequency of substance use, the patients with a higher frequency of substance use were 3–11 times more likely to have NA (59), especially during the pandemic. During the lockdown, patients with higher frequency of drug use were unable to obtain drugs, which intensified their NA (60). In contrast, a few studies did not find such an association, possibly because multiple drug abuse is an important confounding factor (61, 62). Therefore, the characteristics of substance use have a deep-rooted impact on patients with SUDs. In our study, a more important finding was that the impulsivity to use substances was positively correlated with NA in these patients during the pandemic, which is consistent with our previous hypothesis that impulsivity is a powerful predictor of NA in elderly patients with SUDs during the pandemic (37), as greater impulsivity indicated more NA. Previous studies have shown that impulsivity is a susceptible factor in many emotional problems, including NA (63, 64). In fact, it has been reported that the pressure caused by social isolation in response to COVID-19 triggered greater and more frequent cravings and impulsivity for drugs or alcohol in elderly patients with SUDs, which has led to NA and even relapse (65, 66). This is basically consistent with our findings. Meanwhile, there is a growing body of evidence that NA and impulsivity interact in some way, which may provide a hint for developing strategies for the prevention and treatment of drug abuse (67). Therefore, reducing impulsivity in the elderlies with SUDs during the COVID-19 pandemic is crucial for their treatment (68).

Another important finding in this study was that craving during the pandemic was also positively correlated with NA in elderlies with SUDs. This is consistent with our previous hypothesis that craving is a stable predictor of NA in SUDs (36, 69); in other words, patients with greater cravings are likely to have more NA (70). In fact, in most ecological momentary assessment studies, NA was found to be positively correlated with various substance cravings and substance use (71). Many theories of drug dependence and addiction, including negative reinforcement models, such as the self-medication hypothesis (72), suggest that the avoidance of NA plays an important role in the initiation and maintenance of addictive behavior. Our findings also indirectly support that substance abuse involves a common physiological mechanism, i.e., NA forms a negative reinforcement on the use of almost all substances use, leading to an increase in substance use (73). COVID-19 and the subsequent social isolation have triggered NA such as stress, depression, and anxiety, which increased the cravings and consumption of addictive drugs. Therefore, during the severe period of the pandemic, it is necessary to provide psychological counseling for elderly patients with SUDs, proactively treat their NA, and improve drug management after the pandemic to prevent drug (legal or illegal) abuse (22). In addition, taking a break from the news and social media can indirectly help treatment and prevent relapse (13).

## Limitations

There are some limitations in this study. Firstly, only patients aged 50 and over were enrolled, which may affect the generalization of the results. Secondly, the cross-sectional design precluded us from conducting a longitudinal analysis of the relationship between cravings, impulsivity, and NA in this population. Therefore, further follow-up studies are needed. Finally, this study did not assess many other NA related factors, such as stress. Despite these limitations, we believe that this study has the potential to contribute to the field of SUDs in the elderlies, especially with regard to NA.

## Conclusion

Substance abuse in the elderlies has become a worldwide concern during the COVID-19 pandemic, and the treatment and prevention of recurrence are also a challenge for clinicians. This study presented the relationship between NA and various factors in elderly patients with SUDs, and pointed out the significance of routine screening for NA in such patients. We suggest that early diagnosis and treatment of problems of NA and assessment of its related factors may help to reduce recurrence in elderlies with SUDs.

## Data Availability Statement

The data analyzed in this study is subject to the following licenses/restrictions: All data in the current study was stored in the PI's affiliation, and is available from the corresponding authors on reasonable request and with completion of data user agreement. Requests to access these datasets should be directed to TL; liutieqiao123@csu.edu.cn.

## Ethics Statement

The studies involving human participants were reviewed and approved by the Ethics Committee of The Second Xiangya Hospital of Central South University. The patients/participants provided their written informed consent to participate in this study.

## Author Contributions

TL supervised and designed this study. YW, XW, and QY collected the data. JZ, QianW, YL, QiuW, and JT analyzed and interpretation of data. QianW and YW wrote the first draft of the manuscript. TL, XZ, HW, CG, YZ, WY, and YW revised it critically for important intellectual content. All co-authors revised and approved the version to be published.

## Conflict of Interest

The authors declare that the research was conducted in the absence of any commercial or financial relationships that could be construed as a potential conflict of interest.
